# Contemporary Indications for Bioresorbable Scaffolds for Infrapopliteal Peripheral Artery Disease: An International Expert Consensus

**DOI:** 10.1016/j.jscai.2026.105458

**Published:** 2026-06-18

**Authors:** Maxime Dubosq-Lebaz, Peter A. Schneider, Nathan W. Watson, Marianne Brodmann, John H. Rundback, Eric A. Secemsky

**Affiliations:** a Department of Cardiovascular Research, Richard A. and Susan F. Smith Center for Outcomes Research, Beth Israel Deaconess Medical Center, Harvard Medical School, Boston, Massachusetts; b Division of Vascular and Endovascular Surgery, University of California, San Francisco, California; c Department of Medicine, Brigham and Women’s Hospital, Boston, Massachusetts; d Division of Angiology, Department of Internal Medicine, Medical University, Graz, Austria; e Advanced Interventional and Vascular Services, Teaneck, New Jersey

**Keywords:** below-the-knee disease, chronic limb-threatening ischemia, drug-eluting resorbable scaffold, endovascular therapy, expert consensus, peripheral artery disease

## Abstract

**Background:**

Endovascular treatment of below-the-knee disease in chronic limb-threatening ischemia remains challenging, with limited durability of balloon angioplasty and constraints associated with permanent metallic implants. Drug-eluting resorbable scaffolds (DRS) provide temporary vessel support with local drug delivery followed by bioresorption, representing a promising alternative. However, the optimal clinical scenarios for DRS use remain undefined. This study aimed to establish consensus on DRS use in infrapopliteal revascularization.

**Methods:**

A multidisciplinary steering committee developed a structured questionnaire addressing lesion characteristics, patient-level clinical factors, and health-system considerations. Using the RAND/UCLA Appropriateness Method and a modified Delphi process, 114 clinical scenarios were evaluated by an international panel of experts in vascular surgery, interventional cardiology, interventional radiology, and vascular medicine. Experts rated each scenario using a 5-point Likert scale. Consensus strength was defined as strong (>80% agreement), partial (60% to 80%), or absent (<60%).

**Results:**

Thirty-five international experts completed the survey. Strong consensus was achieved for 44 scenarios (38.6%), partial consensus for 35 (30.7%), and no consensus for 35 (30.7%). Strong agreement supported DRS use in patients with CLTI (Rutherford 4-6), vessel diameter ≥3 mm, lesions ≤60 mm, and following adequate vessel preparation, particularly in centers with high procedural expertise and advanced imaging capabilities. Partial consensus emerged for longer or moderately complex lesions and certain clinical contexts. Lack of consensus was observed in small-vessel disease, complex bifurcations, long-segment disease requiring multiple scaffolds, thrombotic lesions, and scenarios involving interruption of antiplatelet therapy.

**Conclusions:**

This consensus defines appropriate clinical scenarios for BTK DRS use, while areas of disagreement identify evidence gaps and research priorities as scaffold technologies evolve.

## Introduction

Chronic limb-threatening ischemia (CLTI) carries a high risk of limb loss, largely driven by involvement of the below-the-knee (BTK) arteries. Endovascular treatment of BTK disease remains challenging, with limited durability of balloon angioplasty, particularly in long, calcified, or multilevel lesions. Off-label coronary stents have been used to manage angioplasty failure but are constrained by compression, in-stent restenosis (ISR), and occlusion. Drug-eluting resorbable scaffolds (DRS) were developed to address these limitations by providing temporary vessel support with local drug delivery followed by bioresorption. In addition, the presence of a permanent metallic implant may limit future therapeutic options for lesion re-intervention, particularly in the setting of recurrent disease. Early clinical experience with limus-eluting resorbable platforms supports the feasibility, safety, and midterm patency of DRS in selected BTK lesions.^1^, ^2^, ^3^, ^4^, ^5^, ^6^ However, the optimal role of DRS in clinical practice remains incompletely defined, particularly regarding patient and lesion selection, vessel preparation strategies, and required expertise. In the absence of guideline recommendations and amid heterogeneous global adoption, we conducted an international multidisciplinary consensus to define clinical scenarios in which DRS use may be considered appropriate, uncertain, or inappropriate for BTK disease.

## Methods

### Steering committee formation

A multidisciplinary steering committee composed of vascular surgeons, interventional radiologists, and interventional cardiologists with extensive experience in BTK endovascular therapy led the development of this consensus (Supplemental Table S1). The committee defined the scope of the project, supervised methodological decisions, constructed and refined the questionnaire, and validated the final interpretation of results.

### Identification of key domains

Through structured meetings and appraisal of the available evidence, the steering committee identified 3 overarching domains considered essential to decision-making regarding DRS use in BTK disease. These domains included lesion and procedural characteristics, patient-level clinical factors, and payor and health-system considerations. This framework guided the development of the questionnaire. Patient-level clinical factors included comorbid conditions (such as diabetes and dialysis dependence), severity of ischemia (Rutherford classification), antiplatelet therapy adherence and bleeding risk, and surgical candidacy. Health-system considerations encompassed institutional expertise, annual procedural volume, availability of advanced imaging modalities (including intravascular ultrasound), access to hybrid or endovascular suites, and the presence of structured postprocedural surveillance programs.

### Questionnaire development

Statements were designed to reflect real-world clinical decision points for DRS implantation. Each domain was subdivided into key categories, including calcification, lesion length, chronic total occlusions, bifurcation anatomy, vessel preparation, antiplatelet therapy adherence, dialysis status, bypass candidacy, operator experience, procedural volume, and institutional capabilities. These were translated into 114 discrete, single-dimension clinical scenarios to minimize ambiguity. Each scenario was designed to reflect a single, clearly defined clinical situation by combining specific anatomical, clinical, and procedural variables, in order to minimize ambiguity and allow consistent interpretation across panelists (Supplemental Table S2). All items were iteratively revised by the steering committee to ensure clarity and consistency. Each scenario was constructed to isolate specific combinations of these variables, allowing the assessment of DRS appropriateness across clearly defined clinical, anatomical, and organizational contexts. The primary outcome assessed was the level of appropriateness for DRS use within each scenario, as rated by the expert panel.

### Survey programming and electronic administration

The final questionnaire was administered using Research Electronic Data Capture (REDCap), a secure web-based platform. Panelists accessed the survey via individualized secure links and completed all ratings independently. The project was reviewed and deemed exempt from institutional review board oversight as it did not constitute human subject research

### Expert panel selection

A total of 52 international experts from North America, Europe, Asia, and the Pacific were nominated by the steering committee. Thirty-five experts completed the full questionnaire, spanning vascular surgery, interventional radiology, interventional cardiology, and vascular medicine. All responses were anonymous.

### Rating process

Experts rated each scenario on a 5-point Likert scale ranging from “rarely appropriate” to “appropriate.” Ratings were collected electronically through REDCap and categorized for analysis as rarely/not appropriate, uncertain, or appropriate/probably appropriate.

### Modified Delphi consensus procedure

A modified Delphi process was conducted. In the first round, all experts rated each of the 114 scenarios independently. The steering committee aggregated responses and generated structured feedback summarizing median scores and rating distributions. No synchronous meeting or group discussion occurred to preserve independence of judgment and minimize hierarchical influence.

### Consensus definitions

Consensus strength was defined a priori according to the RAND/UCLA Appropriateness Method. Consensus categories were determined based on the degree of agreement among expert ratings. Consensus was defined as the proportion of ratings falling within the modal appropriateness category.

Strong consensus was assigned when ratings demonstrated high consensus (>80%) within a single appropriateness category. Partial consensus corresponded to intermediate consensus (60% to 80%), whereas no consensus reflected low consensus (<60%). Final classifications were based on second-round rating distributions.

### Data analysis

For each item, the distribution of responses, modal appropriateness category, and consensus strength were calculated. Descriptive analyses were used to evaluate patterns of agreement across domains, following the methodological framework defined at project initiation. The analysis therefore focused on the distribution of appropriateness ratings across predefined domains rather than clinical outcomes. The overall framework integrating lesion characteristics, patient-level factors, and health-system considerations, along with appropriateness classification, is summarized in the Central Illustration.

**Central Illustration:**
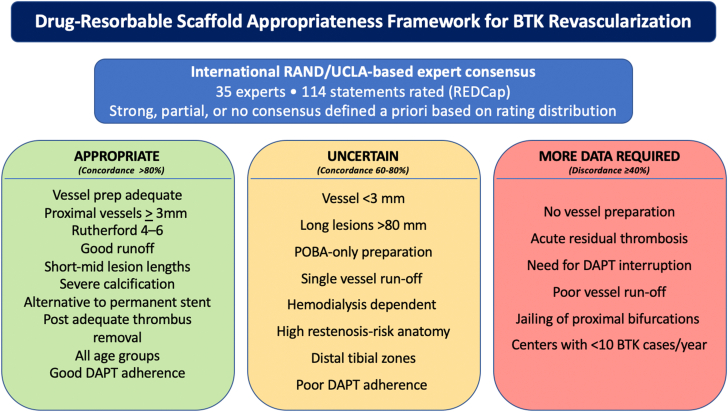
**Drug-resorbable scaffold appropriateness framework for BTK revascularization.** Summary of expert consensus derived from a RAND/UCLA-based methodology across 114 clinical scenarios rated by 35 international experts. Clinical situations are categorized as appropriate, uncertain, or requiring further evidence based on predefined agreement thresholds. The framework integrates lesion characteristics, patient-level factors, and health-system considerations to guide the use of drug-resorbable scaffolds in infrapopliteal interventions. BTK, below-the-knee; DAPT, dual antiplatelet therapy; POBA, plain old balloon angioplasty.

## Results

### Consensus overview

A total of 114 statements were evaluated by 35 experts using the RAND/UCLA Appropriateness Methodology. Strong consensus, defined by discordance below 20%, was achieved for 44 statements (38.6%), most commonly in scenarios associated with clear clinical benefit or standardized procedural pathways. Partial consensus, corresponding to 20% to 39% discordance, was observed for 35 statements (30.7%) and generally reflected context-dependent scenarios, such as DRS use in moderately calcified lesions after intravascular lithotripsy, whereas appropriateness varied according to operator experience, vessel compliance, and the quality of vessel preparation. No consensus, defined by discordance of 40% or greater, was identified for 35 statements (30.7%), predominantly involving anatomically complex lesions, high-risk clinical presentations, or technically uncertain scenarios that remain underrepresented in existing DRS experience.

### Lesion and procedural characteristics

#### Strong consensus (consensus >80%)

Strong consensus supported DRS use in favorable anatomical and procedural settings consistent with those evaluated in the LIFE-BTK trial, particularly when adequate vessel preparation was achieved.^6^ Appropriateness was agreed upon for lesion lengths up to 60 mm, proximal tibial lesions with vessel diameter ≥3 mm, and Rutherford class 4-6 ischemia. High agreement also supported DRS implantation following effective vessel preparation, including intravascular lithotripsy, atherectomy, thrombectomy with complete thrombus removal, and balloon predilatation alone, reflecting confidence in DRS performance when luminal gain and vessel compliance were optimized. Consensus further supported use in moderately to severely calcified lesions after appropriate preparation, TASC C lesions with good runoff, TASC D lesions after successful recanalization, and bifurcations without relevant side-branch involvement.

#### Partial consensus (consensus 60%-80%)

Partial consensus was observed in scenarios where procedural success was more context-dependent, including longer lesions exceeding 60 mm, more complex calcified disease requiring atherectomy with adjunctive therapy, moderately calcified lesions without formal vessel preparation, and selected bifurcation patterns with a dominant branch. Partial agreement also emerged regarding combined DRS and DCB strategies and operator-related factors, reflecting variability in technical experience, familiarity with vessel preparation strategies, and regional differences in device availability.

#### No consensus (discordance ≥40%)

Lack of consensus was achieved for anatomically challenging scenarios, including small-vessel disease (<3 mm), distal peroneal or anterior tibial targets, long-segment disease requiring multiple scaffolds, scaffold-in-stent strategies, jailing of branches in tibioperoneal trunk lesions, or use in ISR and thrombotic disease. These scenarios were characterized by limited supporting evidence and technical uncertainty, contributing to substantial variability in expert opinion.

### Patient-level clinical factors

#### Strong consensus (consensus >80%)

Strong consensus favored DRS use in patients with CLTI (Rutherford 4-6) including gangrene and partial wound healing. Agreement was also high for DRS in patients with good distal runoff. There was consensus that DRS remained a key strategy among patients with excellent dual antiplatelet therapy adherence, those who underwent recent coronary stenting, and those on triple therapy or requiring oral anticoagulation. Broad consensus also supported DRS across age categories—from patients under 50 to older patients with comorbidities—provided clinical context was favorable.

#### Partial consensus (consensus 60%-80%)

Partial consensus characterized scenarios involving Rutherford class 1-3, and Rutherford 6 in dialysis patients without high likelihood of limb salvage. Furthermore, there was partial consensus for patients with preserved GSV where bypass may be an alternative and patients with prior failed interventions (including restenosis or failed bypass).

#### No consensus (discordance ≥40%)

No consensus was observed in patients at high bleeding risk, those requiring interruption of dual antiplatelet therapy, dialysis patients with diffuse medial calcinosis, patients unwilling to take antiplatelet therapy, or those with thrombotic disease or known thrombophilia.

### Health-system and organizational factors

#### Strong consensus (consensus >80%)

The panel strongly agreed that DRS use is appropriate in high-volume centers (>30 cases/y), centers with endovascular-only suites or hybrid rooms, centers with full imaging capabilities and surgical backup, and facilities with structured postprocedural surveillance. Experts also endorsed DRS as a clinically meaningful alternative to permanent metallic stents, particularly if cost-effectiveness and reduced clinically driven target lesion revascularization are demonstrated, with additional strong consensus supporting DRS use in centers with intravascular ultrasound capability. These elements were viewed as markers of institutional readiness, ensuring high-quality vessel preparation, accurate device implantation, and reliable longitudinal follow-up.

#### Partial consensus (consensus 60%-80%)

Partial consensus surrounded operator training requirements, institutional volume thresholds, and centers lacking comprehensive surgical or imaging infrastructure. There was also partial agreement on considering DRS appropriate only after operator certification, highlighting institutional variability in defining procedural competency for these devices.

#### No consensus (discordance ≥40%)

No consensus was reached regarding restriction of DRS use based on reimbursement environment or use in low-volume centers, highlighting heterogeneity in health-system organization and financing across regions.

### Summary of consensus patterns

Across domains, strong consensus consistently supported DRS use in adequately prepared lesions, Rutherford 4-6 CLTI, vessel diameter ≥3 mm, preserved runoff, and treatment within experienced, well-resourced centers. Partial consensus clustered around intermediate-risk anatomical and clinical scenarios, defined as lesions with greater anatomical complexity (including longer lesion length >60 mm, moderate calcification, or bifurcation involvement) and clinical contexts associated with increased uncertainty regarding procedural success or long-term durability.

## Discussion

This international multidisciplinary consensus provides an expert consensus-based framework regarding the use of DRS for infrapopliteal disease based on the currently available data and technology, integrating expert interpretation of the emerging DRS evidence and real-world clinical experience. Although DRS have been evaluated in several early studies, including prospective cohorts, observational series, and randomized trials, the optimal role of DRS in clinical practice remains in evolution.

Early infrapopliteal studies demonstrated that DRS are technically feasible and associated with acceptable patency and limb salvage in selected patients while avoiding a permanent implant. Stabile et al^4^ reported favorable procedural success and 12-month outcomes using a biodegradable biolimus-eluting scaffold in tibial arteries. Huizing et al^7^ later published a pooled infrapopliteal analysis of an everolimus-eluting bioresorbable platform showing encouraging vessel patency and low amputation rates through 2 years. Nasel et al^8^ demonstrated long-term healing and complete bioresorption with a sirolimus-eluting tyrosine-based scaffold. Additional studies by Dia et al^2^ and Kum et al^3^ confirmed the feasibility and acceptable safety profile of sirolimus- and everolimus-based resorbable platforms in real-world CLTI populations, including complex tibial lesions and mixed wound phenotypes. These early data culminated in the LIFE-BTK randomized trial, which demonstrated superior patency, reduced clinically driven target lesion revascularization, and minimal adverse events compared with balloon angioplasty, supporting the role of temporary scaffolding in appropriately selected patients and leading to regulatory approval of the ESPRIT DRS in the USA and other regions.^6^^,^^9^ Importantly, several of the anatomical factors identified in this analysis, including vessel diameter, lesion length, and degree of calcification, are known to influence outcomes across all endovascular strategies in BTK disease and are not specific to DRS. These variables should therefore be interpreted as general determinants of procedural success rather than DRS-specific predictors.

The patterns observed in this consensus closely mirror the available clinical evidence. Strong agreement was reached for anatomical and procedural settings consistent with prior DRS studies, including short to intermediate lesion lengths, adequate vessel diameter, preserved distal runoff, and optimized vessel preparation. The emphasis placed by experts on vessel preparation aligns with procedural strategies reported in DRS cohorts and reflects broader experience with metallic stent implantation in infrapopliteal disease.

Areas of partial or no consensus largely correspond to clinical and anatomical scenarios where evidence remains limited. Long lesions, small vessel diameter, severe calcification without vessel preparation, bifurcation disease, multiscaffold strategies, and ISR have been underrepresented in existing DRS trials and registries, explaining the observed variability in expert opinion. Similarly, uncertainty regarding DRS use in dialysis patients, those at high bleeding risk, or those requiring interruption of antiplatelet therapy reflects biological and management considerations not addressed in current studies and highlights priorities for future investigation. Moreover, DRS technologies are not uniform, and their performance may vary substantially across platforms. Differences in scaffold composition, polymer characteristics, drug formulation, coating processes, and sterilization methods may influence both acute mechanical behavior and long-term vascular response. As such, a class effect cannot be assumed, and specific platforms may be more appropriate for certain anatomical or clinical scenarios. As additional data emerge, further differentiation between DRS technologies may refine their respective indications and expand their role in BTK interventions.

Lastly, this consensus document highlights procedural and organizational determinants that influence real-world use of DRS. Published studies were largely conducted in high-volume centers with access to advanced imaging and structured surveillance pathways, which matches the strong agreement favoring DRS use in experienced institutions. Conversely, no consensus was reached for low-volume centers or environments lacking comprehensive imaging or surgical backup, emphasizing that successful DRS adoption requires not only an appropriate lesion and patient profile but also institutional readiness. These findings should be interpreted in light of the expert-based nature of this consensus, which may favor the use of DRS in experienced centers. Although procedural outcomes are often optimized in such settings, this should not necessarily restrict the adoption of DRS to select institutions, but rather underscores the importance of adequate operator training, procedural expertise, and institutional resources to ensure safe and effective implementation.

Overall, this consensus does not aim to establish prescriptive treatment guidelines but rather to contextualize current evidence within expert practice. Areas of agreement define scenarios in which DRS may be reasonably adopted in contemporary BTK interventions, whereas areas of disagreement delineate gaps where further research and device innovation are needed. As next-generation scaffold technologies continue to evolve, ongoing data generation will be essential to refine patient selection and expand the role of DRS across the spectrum of CLTI.

In addition to clinical considerations, economic factors are likely to play a central role in the adoption of DRS. Device cost and variability in reimbursement across healthcare systems may significantly influence real-world utilization. In particular, the development of evidence supporting differentiated reimbursement strategies may be important to ensure sustainable adoption, especially when considering alternative technologies such as drug-eluting stents in younger patient populations. Alignment between clinical benefit, cost-effectiveness, and reimbursement frameworks will be essential to support broader implementation.

## Conclusion

In this international multidisciplinary consensus, experts identified scenarios in which DRS are considered appropriate for infrapopliteal revascularization, particularly in patients with advanced ischemia, favorable anatomy, adequate vessel preparation, and treatment within experienced centers. Persistent uncertainty regarding small-vessel disease, complex bifurcations, long lesions, thrombosis, and limited antiplatelet tolerance highlights gaps in the current evidence base and priorities for future research. As ongoing trials and next-generation technologies continue to evolve, this consensus provides a structured, practice-oriented framework to support the safe and rational adoption of DRS for below-the-knee interventions.

## CRediT authorship contribution statement

**Maxime Dubosq-Lebaz:** Conceptualization, Data curation, Investigation, Writing – original draft. **Peter A. Schneider:** Conceptualization, Formal analysis, Funding acquisition, Project administration, Validation, Writing – review & editing. **Nathan W. Watson:** Methodology, Visualization, Writing – review & editing. **Marianne Brodmann:** Formal analysis, Supervision, Writing – review & editing. **John H. Rundback:** Resources, Supervision, Writing – review & editing. **Eric A. Secemsky:** Conceptualization, Funding acquisition, Investigation, Methodology, Project administration, Supervision, Validation, Writing – review & editing.

## Declaration of competing interest

Peter Schneider is a consultant to Surmodics, Medtronic, Boston Scientific, Cagent, Acotec, Abbott, Endologix, Shockwave Medical, Stryker, Healthcare Inroads, Inari, and BD. Marianne Brodmann is a consultant for Abbott, R3 Vascular, and Stentit. John Rundback is a consultant for Abbott, AngioDynamics, BD Bard, Boston Scientific, Cordis, Inari Medical, Medtronic, and Philips; a board member of the CLI Global Society; honoraria from Abbott, AngioDynamics, BD Bard, Boston Scientific, Medtronic, and Philips; and owns stocks/options in Aveera, Protexa, Summa, Evident, and Inquis. Eric Secemsky receives funding from NIH/NHLBI K23HL150290 and the US Food & Drug Administration; has institutional grants from Abbott/CSI, BD, Boston Scientific, Cook, Medtronic, and Philips; and receives speaking/consulting fees from Abbott/CSI, BD, BMS, Boston Scientific, Cagent, Conavi, Cook, Cordis, Endovascular Engineering, Gore, InfraRedx, Medtronic, Philips, RapidAI, Rampart, Shockwave Medical, Siemens, Teleflex, Terumo, Thrombolex, VentureMed, and Zoll. Maxime Dubosq-Lebaz and Nathan Watson reported no financial interests.

## Supplementary material

Supplemental Appendix 1 Chairs and Respondents of DRS Consensus Statement. Supplemental Appendix 2. Clinical Scenarios Evaluated in the RAND/UCLA consensus.

## Footnotes

To access the supplemental material accompanying this article, visit the online version of the *Journal of the Society for Cardiovascular Angiography & Interventions* at 10.1016/j.jscai.2026.105458.
