# Supplementary material for: Contemporary Indications for Bioresorbable Scaffolds for Infrapopliteal Peripheral Artery Disease: An International Expert Consensus
Source: J Soc Cardiovasc Angiogr Interv. 2026 Jun 18;5(7):105458. doi: 10.1016/j.jscai.2026.105458 (PMC13400105; doi:10.1016/j.jscai.2026.105458)
Supplement: Supplemental Appendix 1 — Chairs and Respondents of DRS Consensus Statement. Supplemental Appendix 2. Clinical Scenarios Evaluated in the RAND/UCLA consensus. [file mmc1.docx]

**Supplemental Appendix 1:** Chairs and Respondents of DRS Consensus Statement

| **Physician** | **Specialist** | **Department** | **Country** |
| --- | --- | --- | --- |
| **Experts committee** | | |  |
| Eric A. Secemsky | Interventional cardiologist | Beth Israel Deaconess Medical Center | USA |
| Peter A. Schneider | Vascular surgeon | University of California, San Francisco | USA |
| Marianne Brodmann | Angiologist | Medical University of Graz | Austria |
| John Rundback | Interventional radiologist | Holy Name Medical Center | USA |
| Maxime Dubosq-Lebaz | Vascular surgeon | Beth Israel Deaconess Medical Center | France |
| Nathan W. Watson | Interventional cardiologist | Brigham and Women’s Hospital | USA |
| **Voting Experts** | | | |
| Ajit Rao | Vascular surgeon | Mt. Sinai | USA |
| Andrew Holden | Interventional radiologist | Auckland City Hospital | New Zealand |
| Athanasios Saratzis | Vascular surgeon | University of Leicester & NIHR BRC | United Kingdom |
| August Ysa | Vascular surgeon | Hospital de Cruces. Barakaldo | Spain |
| Brian DeRubertis | Vascular surgeon | New York-Presbyterian (NYP) \| Weill Cornell Medicine | USA |
| Daniel van den Heuvel | Interventional radiologist | St. Antonius Hospital Nieuwegein | The Netherlands |
| Danielle R. Bajakian | Vascular surgeon | New York-Presbyterian, Columbia University Irving Medical Center, | USA |
| Dierk Scheinert | Angiology/Vascular Medicine | Park Krankenhaus | German |
| Ehrin Armstrong | Interventional cardiologist | Advanced Heart and Vein Center | USA |
| Esau Martinez | Vascular surgeon | Mataró Hospital Angiology and Vascular Surgery Unit. | Spain |
| Fabrizio Fanelli | Interventional radiologist | "CAREGGI" University Hospital - University of Florence | Italy |
| Gerd Grözinger | Interventional radiologist | University Hospital Tübingen | Germany |
| Gianmarco De Donato | Vascular surgeon | University of Siena | Italy |
| Gilles Goyault | Interventional radiologist | Clinique Rhena, Strasbourg, France | France |
| Hany Zayed | Vascular surgeon | King's College London and Guy's and St. Thomas' Hospital | United Kingdom |
| James McCaslin | surgeon | The Newcastle upon Tyne Hospitals | United Kingdom |
| Jen-Kuang Lee | Interventional cardiologist | National Taiwan University Hospital | Taiwan |
| Joakim Nordanstig | Vascular surgeon | University of Gothenburg, Sweden | Sweden |
| Kumar Madassery | Interventional cardiologist | Rush University Medical Center | USA |
| Leigh Ann O'Banion | Vascular surgeon | UCSF Fresno | USA |
| Lorenzo Patrone | Interventional radiologist | Usl Toscana Centro, San Giovanni di Dio, Hospital, Florence, Italy | Italy |
| Mahmood Razavi | Interventional radiologist | Providence Medical Foundation | USA |
| Marta Lobato | Vascular surgeon | Hospital de Cruces. Barakaldo | Spain |
| Mehdi Shishehbor | Interventional cardiologist | UH Cleveland Medical Center | USA |
| Michael Lichtenberg | Angiology/Vascular Medicine | Arnsberg Clinic | Germany |
| Michael Siah | Vascular surgeon | UT Southwestern | USA |
| Prakash Krishnan | Interventional cardiologist | ICANH School of Medicine | USA |
| Raghuram Lakshminarayan | Interventional radiologist | Hull University Teaching Hospitals NHS Trust | United Kingdom |
| Ralf Langhoff | Angiology/Vascular Medicine | Sankt Gertrauden Krankenhaus Berlin | Germany |
| Ramon Varcoe | Vascular surgeon | Prince of Wales Private Hospital | Australia |
| Robert Lookstein | Interventional Radiology | Icahn School of Medicine at Mount Sinai, New York | USA |
| Sabine Steinert | Angiology/Vascular Medicine | Medical University of Vienna | Austria |
| Sameh Sayfo | Interventional cardiologist | Baylor Scott & White The Heart Hospital - Plano | USA |
| Skyi Pang | Vascular surgeon | Queen Mary Hospital | Hong Kong |
| Steven Kum | Vascular surgeon | Changi General Hospital | Singapour |
| Tania Guzman | Vascular surgeon | Sheikh Shakhboot Medical City | UAE |
| Tatsuya Nakama | Interventional cardiologist | Tokyo Bay Medical Center, Chiba, JP | Japan |
| Uei Pua | Interventional radiologist | Department of Diagnostic Radiology, Tan Tock Seng Hospital | Singapour |
| Venita Chandra | Vascular surgeon | Stanford University | USA |
| Yann Gouëffic | Vascular surgeon | Groupe Hospitalier Paris Saint Joseph, Paris, France | France |
| Zola Ndandu | Interventional cardiolo | University of Florida St Johns/Flagler | USA |

**Supplemental Appendix 2. Clinical Scenarios Evaluated in the RAND/UCLA consensus**

**PARTICIPANT CHARACTERISTICS**

1. **Please indicate your medical specialty:**

- Vascular Surgeon
- Interventional Cardiologist
- Interventional Radiologist
- Angiologist / Vascular Medicine Specialist

**I. LESION / PROCEDURAL CHARACTERISTICS**

A. Calcification

2. Use of DRS in non-calcified lesions without vessel preparation

3. Use of DRS in non-calcified lesions with vessel preparation

4. Use of DRS in mild to moderately calcified lesions without vessel preparation

5. Use of DRS in moderately calcified lesions after POBA alone

6. Use of DRS in moderately calcified lesions after IVL

7. Use of DRS in severely calcified lesions without vessel preparation

8. Use of DRS in severely calcified lesions after atherectomy + POBA

9. Use of DRS in severely calcified lesions after IVL + POBA

A1. Vessel Preparation – Non-Calcified Lesions

10. No vessel preparation

11. Plain balloon predilatation

12. Scoring or cutting balloon

A2. Vessel Preparation – Mild to Moderate Calcification

13. No vessel preparation

14. Plain balloon predilatation

15. Scoring or cutting balloon

16. Intravascular lithotripsy (IVL)

17. Atherectomy (orbital or rotational)

A3. Vessel Preparation – Severe Calcification

18. Plain balloon predilatation

19. Scoring or cutting balloon

20. Intravascular lithotripsy (IVL)

21. Atherectomy (orbital or rotational)

22. Combined IVL + atherectomy

B. TASC Classification

23. Use of DRS in TASC A lesions

24. Use of DRS in TASC B lesions

25. Use of DRS in TASC C lesions with good runoff

26. Use of DRS in TASC C lesions without adequate preparation

27. Use of DRS in TASC D lesions post-successful revascularization

C. Lesion Length

28. Use of DRS in lesion <30 mm

29. Use of DRS in lesion between 40–60 mm

30. Use of DRS in lesion 60–80 mm after POBA alone

31. Use of DRS in lesion 60–80 mm after atherectomy + POBA

32. Use of DRS in lesion >80 mm after IVL + POBA

33. Implantation of multiple DRS (3 or more) in long segment disease

D. Thrombosis

34. Use of DRS in a patient with a history of lower extremity arterial thrombosis (none currently present)

35. Use of DRS in the presence of mural thrombus

36. Use of DRS after successful recanalization and thrombus resolution

37. Use of DRS post-thrombectomy with successful recanalization and thrombus removal

38. Use of DRS in a patient with known thrombophilia

E. Bifurcation

39. Use of DRS at tibial bifurcation sites

40. Use of DRS in a bifurcation with a single dominant branch

41. Use of DRS in TPT bifurcation using T-stenting or culotte technique

42. Use of DRS just proximal to a BTK bifurcation with no side branch disease

43. Use of DRS just proximal to a BTK bifurcation with side branch disease

44. Use of DRS in a single tibioperoneal trunk, jailing one branch

F. Vessel Preparation

45. DRS after plain balloon pre-dilatation

46. DRS after IVL

47. DRS after atherectomy (directional or rotational)

48. DRS after combined atherectomy + DCB

49. DRS without any prior vessel preparation

G. Concomitant Drug-Coated Balloon Use

50. Combined use of DRS and DCB in the same lesion

51. DCB applied just distal to the DRS

52. DCB on adjacent contiguous segment to the DRS

53. Full DCB coverage before DRS implantation

54. Avoiding DCB when DRS is used

H. In-Stent Restenosis

55. DRS in ISR following prior DES

56. DRS in ISR following prior BMS

57. Use of DRS within a previously implanted metallic stent

58. DRS should be avoided in ISR

I. Proximal vs Distal Lesion

59. DRS in proximal anterior tibial artery lesions with vessel diameter ≥3 mm

60. DRS in distal anterior tibial artery lesions with vessel diameter <3 mm

61. DRS in proximal peroneal lesions with good runoff and vessel diameter ≥3 mm

62. DRS in distal peroneal lesions <3 mm

63. DRS in pedal or plantar artery lesions regardless of size

**II. PATIENT PROFILE**

A. Rutherford Classification

64. DRS in Rutherford class 1–3 (claudication)

65. DRS in Rutherford class 4 (rest pain)

66. DRS in Rutherford class 5 (ulceration)

67. DRS in Rutherford class 5 with partial wound healing

68. DRS in Rutherford class 6 (gangrene)

B. Dialysis Status

69. DRS in a dialysis patient with good distal runoff

70. DRS in dialysis patient with diffuse medial calcinosis

71. DRS in dialysis patient with Rutherford 5 disease

72. DRS in dialysis patient with Rutherford 6 disease

73. Systematic avoidance of DRS in dialysis patients

C. Age

74. DRS in a patient under 50 years old with no major comorbidities

75. DRS in a patient under 50 years old with major comorbidities

76. DRS in a patient 50–65 years old with no major comorbidities

77. DRS in a patient 50–65 years old with major comorbidities

78. DRS in a patient over 65 years old with no major comorbidities

79. DRS in a patient over 65 years old with major comorbidities

80. Age alone should not limit DRS use if other criteria are favorable

D. Future Bypass Candidacy

81. DRS as a bridge to future distal bypass

82. 2 DRS in patients with no surgical option

83. DRS in a patient with preserved great saphenous vein

84. DRS in segment critical for future bypass anastomosis

E. DAPT Compliance

85. DRS in a patient with excellent adherence to DAPT

86. DRS in a non-adherent patient

87. DRS in a patient at high bleeding risk

88. DRS in a patient post recent coronary stenting

89. DRS in a patient refusing antiplatelet therapy

90. DRS in a patient on chronic oral anticoagulation

91. DRS in a patient on triple therapy

92. DRS in a patient requiring DAPT interruption

**III. PAYER / HEALTH SYSTEM CONSIDERATIONS**

A. Quality of Life Impact

93. DRS to preserve vessel architecture in active patients

94. DRS to reduce repeat interventions in short term

95. DRS to improve healing and avoid surgical wounds

96. DRS to delay surgical bypass

97. DRS as alternative to permanent metallic stent

B. Costs

98. DRS justified by reduced major amputation rate

99. DRS acceptable if CD-TLR is reduced

100. DRS only if reimbursed

101. DRS acceptable if cost effective compared with alternatives

102. DRS limited to trials due to high cost

C. Procedural Volume

103. DRS in centers with <10 annual cases

104. DRS in centers with >30 annual cases

105. DRS limited to expert centers during early adoption

106. DRS use after certified training of operator

107. DRS limited to experienced trialists

D. Procedure Setting

108. DRS in hospital settings with full imaging and surgical backup

109. DRS in private centers with structured follow-up

110. DRS in hybrid rooms with surgical availability

111. DRS in centers equipped with IVUS

E. Restenosis / Reintervention Risk

112. DRS in high restenosis risk lesions

113. DRS in patients with prior failed interventions

114. DRS in patient refusing bypass

115. Strict post-DRS surveillance required to detect restenosis
